# Health Care Provider Perceptions of Consumer-Grade Devices and Apps for Tracking Health: A Pilot Study

**DOI:** 10.2196/mhealth.9929

**Published:** 2019-01-22

**Authors:** Bree Holtz, Kerri Vasold, Shelia Cotten, Michael Mackert, Mi Zhang

**Affiliations:** 1 Department of Advertising and Public Relations College of Communication Arts & Sciences Michigan State University East Lansing, MI United States; 2 Department of Kinesiology Michigan State University East Lansing, MI United States; 3 Department of Media & Information College of Communication Arts & Sciences Michigan State University East Lansing, MI United States; 4 Center for Health Communication Stan Richards School of Advertising & Public Relations University of Texas, Austin Austin, TX United States; 5 Department of Electrical and Computer Engineering College of Engineering Michigan State University East Lansing, MI United States

**Keywords:** physicians, primary care, APRN, nurse practitioners, technology

## Abstract

**Background:**

The use of Web- or mobile phone–based apps for tracking health indicators has increased greatly. However, provider perceptions of consumer-grade devices have not been widely explored.

**Objective:**

The purpose of this study was to determine primary care physicians’ and advanced practice registered nurses’ perceptions of consumer-grade sensor devices and Web- or mobile phone–based apps that allow patients to track physical activity, diet, and sleep.

**Methods:**

We conducted a cross-sectional mailed survey with a random sample of 300 primary care physicians and 300 advanced practice registered nurses from Michigan, USA. Providers’ use and recommendation of these types of technologies, and their perceptions of the benefits of and barriers to patients’ use of the technologies for physical activity, diet, and sleep tracking were key outcomes assessed.

**Results:**

Most of the respondents (189/562, 33.6% response rate) were advanced practice registered nurses (107/189, 56.6%). Almost half of the sample (93/189, 49.2%) owned or used behavioral tracking technologies. Providers found these technologies to be helpful in clinical encounters, trusted the data, perceived their patients to be interested in them, and did not have concerns over the privacy of the data. However, the providers did perceive patient barriers to using these technologies. Additionally, those who owned or used these technologies were up to 6.5 times more likely to recommend them to their patients.

**Conclusions:**

Our study demonstrated that many providers perceived benefits for their patients to use these technologies, including improved communication. Providers’ concerns included their patients’ access and the usability of these technologies. Providers who encountered data from these technologies during patient visits generally perceive this to be helpful. We additionally discuss the barriers perceived by the providers and offer suggestions and future research to realize the potential benefits to using these data in clinical encounters.

## Introduction

### Background

Almost 50% of US adults report having one or more chronic diseases [1]. Chronic disease care accounted for 86% of health care spending in the United States in 2014 [2]. Health risk behaviors, including physical inactivity and poor nutrition, are cited as highly attributable causes for the illness and early death associated with chronic diseases [3]. With less than 50% of US adults meeting physical activity guidelines, and less than 25% of US adults meeting nutrition guidelines [3], behavioral aspects of health are considered a key component to target to reduce chronic disease risk and prevalence. Tracking health indicators is one behavior that is promoted to help address these healthy behavior choices.

Self-care through tracking health indicators has been shown to be successful in the management of many chronic diseases. Patient self-tracking, or recording of health indicators at home, has been used in a variety of situations, including the prediction of events such as migraines, weight control, physical activity patterns, and self-management of blood pressure and blood glucose [4-7]. The popularity of self-tracking has also grown, with almost 70% of US adults tracking a health indicator such as weight, diet, exercise, or health symptoms for themselves or for another individual [8], with similar rates in other countries [9]. In the past, much of this self-tracking has been done through the use of paper diaries [8]. Recently, the use of technology to more accurately track health indicators has increased; technologies such as consumer-grade sensor devices (eg, Fitbit) and Web- or mobile phone-based apps (eg, MyFitnessPal) that allow patients to track physical activity, diet, sleep, and a variety of other factors have proliferated [9]. In fact, the US Food and Drug Administration recently approved Apple Inc’s smartwatch for monitoring the heart (through electrocardiography) and to detect atrial fibrillation [10]. Using these technologies has been associated with positive health outcomes across a wide range of conditions and behaviors, such as diet, physical activity, weight management, and mental health [11,12]. With this increased use of technologies for self-tracking of health behaviors, there are many implications for use of these technology-generated data in the clinical setting.

Much of the previous research has focused on consumer perspectives of these technologies [13]. Studies have examined the benefits to individuals who are healthy and simply want to track and improve their overall lifestyle [14-19]. As for individuals who have a chronic condition (eg, asthma, depression, diabetes), the evidence suggests that tracking may be beneficial [20-24]; however, the technologies’ use in the clinical setting remains limited. Research on primary care providers’ perceptions of information technologies that have been developed to be used as a diary for a specific illness is also limited, with only a handful of studies identified [25-29]. Additionally, 1 study was conducted on perceptions of data displays among both health care providers and laypersons [30]. Much of the research has hypothesized only about provider perspectives based on past experiences with other technologies [31,32]. Understanding primary care providers’ perceptions may yield knowledge that can result in better design and use of these technologies for monitoring and managing patients’ health, and improving their health outcomes.

### Objectives

The objectives of this pilot study were to determine primary care physicians’ and advanced practice registered nurses’ (APRNs) use and perceptions of health tracking technologies. We included APRNs in this study because there is a well-documented shortage of primary care physicians, and APRNs often fulfill the role of a primary care health care provider for many patients [33]. We also sought providers’ perceptions of the usefulness of these technologies on a variety of health issues. Additionally, we examined whether there were differences in perceptions by provider technology use status, as well as any differences between physicians and APRNs.

## Methods

### Study Design and Participants

In a cross-sectional study taking place from July to September 2016, using a random number table, we selected a random sample of 300 primary care physicians and 300 APRNs from office and hospital settings from the entire state of Michigan, USA, from a list purchased from DMD Marketing Corporation (an approved American Medical Association database licensee; Rosemont, IL, USA) and asked them to participate in a mail survey. We mailed providers a study packet that included a welcome letter, the survey, a self-addressed stamped return envelope, and a US $5 gift card to a national coffee chain. Follow-up postcards were sent to individuals who had not returned their original survey after 2 weeks. Past studies have demonstrated that mail surveys provide the best response rate of physicians [34-36]; however, on both the survey and the postcard follow-up, we provided a link to an online version of the survey (Qualtrics, Provo, UT, USA). The study was approved by the Michigan State University Institutional Review Board.

### Data Collection

**Figure 1:**
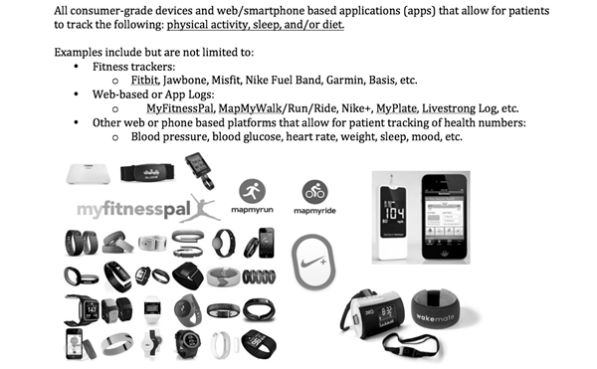
The definitions of consumer-grade sensor devices and Web- or mobile phone-based apps that health care providers were given in the survey.

We developed the survey through a literature review of providers’ perceptions regarding various technologies and patients bringing information to the visits [25,37]. From these studies, we developed items reflecting the themes from the results. We then conducted a small pilot study of the survey with 3 physicians and 2 nurse practitioners. The feedback they provided required us to shorten the survey and, if possible, to provide a larger incentive. The final survey included 25 questions in total pertaining to providers’ use of these technologies (as defined above, consumer-grade sensor devices, such as Fitbit, and Web- or mobile phone-based apps, such as MyFitnessPal), their patients’ use of these technologies, their perceptions of the usefulness of these technologies, and demographic questions. Figure 1 provides the definition of consumer-grade sensor devices and Web- or mobile phone-based apps that the providers were given. We also asked providers about their personal use of these technologies. These were answered on a 5-point Likert-type scale (ranging from “strongly agree” to “strongly disagree”). An additional 9 questions pertained to the usefulness of technologies in specific contexts (eg, physical activity, diet, sleep, medication adherence, goal setting). Demographic and organizational characteristics were also included.

### Statistical Analysis

We calculated descriptive statistics for all variables of interest. We conducted an exploratory factor analysis on the 19 perception statements of the data collected by technologies, barriers to use, and benefits of technologies. We identified 5 factors among 16 of the 19 statements; the remaining statements did not associate with a factor. Table 1 shows the factor analysis loadings. Reliability analysis revealed moderate to high reliability for all 5 factors (alpha≥.69).

We conducted Mann-Whitney *U* tests to determine whether the means of the 5 factors and individual perceptions questions were different for user versus nonuser and physician versus APRN comparisons. We used multinomial logistic regression using 95% confidence intervals to evaluate relationships for the likelihood of recommendation of technologies and perceptions of barriers and benefits. Independent variables for analyses included user status and job status. Dependent variables included likelihood of recommendation of technologies and perceptions of barriers and benefits. For the purpose of logistic regression, we condensed the perception scale categories to three—(1) agreement (“strongly agree” and “agree”), (2) neither agree nor disagree, and (3) disagreement (“strongly disagree” and “disagree”)—and treated them as categorical data within the analysis. All analyses were conducted in IBM SPSS 24.0 statistical software (IBM Corporation).

**Table 1:** Exploratory factor analysis loadings.

Perception statements	Factors				
	Data review	Provider trust of data	Patient’s interest in technologies	Security and liability	Perceived patient barriers
There are worthwhile health benefits from reviewing patient-tracked data	.443^a^	.117	.564	.012	.027
Data from these apps and devices help me manage my patients’ visits	.579^a^	.093	.511	.014	–.081
I want the data from these apps and devices to link with the electronic medical record system	.882^a^	.061	.020	–.131	–.052
I want the data from these apps and devices to link with the patient portal	.860^a^	.055	.015	–.111	–.023
I don’t trust patient-reported data from these apps and devices^b^	.109	.874^a^	.124	–.136	–.076
I don’t trust the data these apps and devices provide^b^	.107	.868^a^	.152	–.151	–.025
My patients are not familiar with tracking using these devices^b^	–.115	–.128	.541^a^	–.135	–.455
The technologies available to my patients are not useful^b^	.018	.475	.644^a^	–.205	.061
My patients are not interested in using technology to track behaviors or health^b^	–.083	.086	.732^a^	–.015	–.084
There is no value to me if my patients use these types of devices^b^	.268	.368	.646^a^	–.061	.140
I am concerned about security and privacy of the data collected from these devices and apps	–.077	–.058	–.111	.789^a^	.032
I am concerned about liability issues when it comes to recommending these devices and apps	–.095	–.107	–.084	.881^a^	.088
I am concerned about liability issues when viewing data as part of an electronic medical record system from these devices and apps	–.083	–.130	.480	.887^a^	.091
My older patients have a harder time with technology	–.058	.021	–.147	–.007	.811^a^
Not everyone has sufficient technological literacy to use these devices and apps	–.046	–.081	.012	.038	.800^a^
Not everyone has sufficient access to these devices and apps	–.105	–.032	.048	.184	.785^a^
Patients do not want to share their data with me because they don’t want me to know the truth about their health^c^	.295	–.344	–.131	.340	.056
I don’t get reimbursed for reviewing these data^c^	.086	–.323	.374	–.070	.245
Patients need to have enough detail to make reviewing tracked data worthwhile^c^	.238	–.120	.232	.001	.362

^a^Statement that loaded under the factor.

^b^Reverse coded.

^c^Removed from analysis because it did not fall within a factor.

## Results

### Sample Description

Respondents (189/562, 33.6% response rate; 38 packets were returned unopened) were primarily female (133/189, 70.4%), white (163/183, 89.1%), and between the ages of 35 and 54 years (96/188, 51.1%). Of the 189 completed surveys, 15 were completed using the Qualtrics link. The sample was divided between physicians (82/189, 43.4%) and APRNs (107/189, 56.6%), and 49.2% of respondents (93/189) reported using these types of technologies (as defined above) themselves. When asked about the typical insurance coverage their patients had, the sample reported that 41.9% of their patients had private insurance, 31.7% had Medicare, 27.5% had Medicaid, and 8.5% were uninsured (mean responses). Table 2 provides more detailed demographic information.

### Provider Perceptions

The 5 factors from the factor analysis of perceptions questions were (1) data review (alpha=.78), defined as providers perceiving these data to be useful in patient encounters and wanting the data to be available through the electronic medical record system (EMR); (2) provider trust of the data (alpha=.79), including questions on the trustworthiness of the data from these technologies; (3) patients’ interest in the technologies (alpha=.69), defined as the providers’ perceptions of how interested in these technologies they believed their patients to be; (4) security and liability (alpha=.9), defined as the providers’ perceptions of the data from these technologies; and (5) perceived patient barriers (alpha=.85), including the providers’ perceptions of their patients’ age, technology literacy, and access to these technologies. In the analysis of factors overall and individual questions pertaining to factors, notable findings included significant differences (*P* value range from <.001 to .02) between users and nonusers for data review, provider trust of data, and patients’ interest in technologies. Additionally, we found only one difference by job status: APRNs were more interested in the linking of patient data to patient portals than physicians were (*P*=.02). Table 3 provides more detailed results of overall perceptions.

**Table 2:** Respondents’ demographic characteristics.

Characteristics		Overall (n=189)	Physicians (n=82)	Advanced practice registered nurses (n=107)
**Sex, n (%)**				
	Male	56 (29.6)	52 (63)	4 (3.7)
	Female	133 (70.4)	30 (36)	103 (96.3)
**Age (years), n (%)**				
	25-34	18 (9.6)	6 (7)	12 (11.2)
	35-54	96 (51.1)	48 (59)	48 (44.9)
	55-64	69 (36.7)	27 (33)	42 (39.3)
	≥65	5 (2.7)	0 (0)	5 (4.7)
**Race/ethnicity, n (%)**				
	White	163 (89.1)	66 (85)	97 (92.4)
	African American	3 (1.6)	1 (1)	2 (1.9)
	Asian	11 (6.0)	9 (12)	2 (1.9)
	Native Hawaiian or Pacific Islander	1 (0.5)	1 (1)	0 (0)
	American Indian or Alaskan Native	2 (1.1)	1 (1)	1 (1.0)
	Other	3 (1.6)	0 (0)	3 (2.9)
**User status, n (%)**				
	Users	93 (49.2)	32 (39)	61 (57.0)
	Nonusers	96 (50.8)	50 (61)	46 (43.0)
**Insurance, mean (SD)**				
	Private	41.9 (22.8)	45.7 (20.3)	38.2 (24.6)
	Medicare	31.7 (18.5)	32.7 (13.3)	30.5 (22.8)
	Medicaid	27.5 (22.0)	20.0 (17.9)	34.9 (28.8)
	Uninsured	8.5 (12.3)	5.6 (5.5)	11.2 (15.9)

**Table 3:** Provider recommendations of tracking technologies and perceptions of technologies by job title and user status^a^.

Independent variable			Overall (n=189)	User (n=93)	Nonuser (n=96)	Physician (n=82)	Advanced practice registered nurses (n=107)
**Recommendation for..., n (%)**							
	Physical activity		124 (65.6)	79 (845)	45 (47)	52 (63)	72 (67.3)
	Diet		118 (62.4)	77 (83)	41 (43)	49 (60)	69 (64.5)
	Sleep		37 (19.6)	26 (28)	11 (12)	14 (17)	23 (21.5)
**Perceptions of..., mean (SD)**							
	**Data review**		3.3 (0.7)	3.5 (0.7)^b^ *P*<.001	3.2 (0.7)	3.3 (0.8)	3.4 (0.7)
		There are worthwhile health benefits from reviewing patient-tracked data	3.9 (0.8)	4.0 (0.8)^b^ *P*=.009	3.8 (0.8)	3.8 (0.9)	4.0 (0.7)
		Data from these apps and devices help me manage my patients’ visits	3.3 (1.0)	3.5 (0.9)^b^ *P*=.002	3.0 (1.0)	3.3 (1.0)	3.2 (1.0)
		I want the data from these apps and devices to link with the electronic medical record system	3.0 (1.0)	3.3 (1.0)^b^ *P*=.005	2.8 (1.0)	2.9 (1.1)	3.1 (1.0)
		I want the data from these apps and devices to link with the patient portal	3.2 (1.0)	3.4 (0.9)^b^ *P*=.003	3.0 (1.0)	3.0 (1.1)^c^ *P*=.02	3.4 (0.9)
	**Provider trust of data**		3.5 (0.8)	3.6 (0.7)^b^ *P*=.007	3.4 (0.8)	3.4 (0.8)	3.6 (0.7)
		I don’t trust patient-reported data from these apps and devices^d^	3.4 (0.8)	3.5 (0.8)	3.4 (0.8)	3.3 (0.8)	3.5 (0.8)
		I don’t trust the data these apps and devices provide^d^	3.6 (0.8)	3.7 (0.8)^b^ *P*=.001	3.4 (0.8)	3.4 (0.8)	3.7 (0.8)
	**Patient’s interest in technologies**		3.6 (0.6)	3.7 (0.5)^b^ *P*<.001	3.4 (0.6)	3.6 (0.6)	3.6 (0.6)
		My patients are not familiar with tracking using these devices^d^	3.1 (0.9)	3.3 (1.0)	3.0 (0.9)	3.3 (0.9)	3.0 (0.9)
		The technologies available to my patients are not useful^d^	3.8 (0.7)	4.0 (0.7)^b^ *P*=.001	3.7 (0.7)	3.8 (0.8)	3.8 (0.6)
		My patients are not interested in using technology to track behaviors or health^d^	3.5 (0.9)	3.7 (0.8)^b^ *P*=.02	3.4 (0.9)	3.5 (0.9)	3.5 (0.9)
		There is no value to me if my patients use these types of devices^d^	3.8 (0.8)	4.0 (0.7)^b^ *P*=.001	3.6 (0.9)	3.8 (0.9)	3.9 (0.7)
	**Security and liability**		2.8 (0.9)	2.7 (0.9)	2.9 (0.9)	2.7 (1.0)	2.9 (0.8)
		I am concerned about security and privacy of the data collected from these devices and apps	2.8 (1.0)	2.7 (1.0)	2.9 (1.0)	2.7 (1.0)	2.9 (1.0)
		I am concerned about liability issues when it comes to recommending these devices and apps	2.7 (1.0)	2.6 (1.0)	2.8 (1.0)	2.6 (1.1)	2.8 (0.9)
		I am concerned about liability issues when viewing data as part of an electronic medical record system from these devices and apps	3.0 (1.1)	2.9 (1.1)	3.1 (1.1)	2.9 (1.2)	3.1 (1.0)
	**Perceived patient barriers**		3.9 (0.7)	3.9 (0.7)	3.9 (0.7)	3.9 (0.7)	3.9 (0.7)
		My older patients have a harder time with technology	3.9 (0.9)	3.9 (0.9)	3.9 (0.8)	4.0 (0.8)	3.8 (0.9)
		Not everyone has sufficient technological literacy to use these devices and apps	3.9 (0.8)	4.0 (0.8)	3.9 (0.8)	3.9 (0.9)	3.9 (0.7)
		Not everyone has sufficient access to these devices and apps	4.0 (0.8)	4.0 (0.8)	4.0 (0.8)	3.9 (0.8)	4.1 (0.7)

^a^Perceptions scale: 1 = strongly disagree, 2 = disagree, 3 = neither agree nor disagree, 4 = agree, 5 = strongly agree.

^b^Significantly different from nonusers; *P*<.05.

^c^Significantly different from advanced practice registered nurses; *P*<.05.

^d^Question was reverse coded.

**Table 4:** Odds ratios (ORs) and 95% CIs for comparisons of user status and job title (N=189).

Independent variable		User vs nonuser^a^, OR (95% CI)	Physician vs advanced practice registered nurses^b^, OR (95% CI)
**Recommendation for...**			
	Physical activity	6.4^c^ (3.2-12.8)	0.8 (0.5-1.5)
	Diet	6.5^c^ (3.3-12.7)	0.8 (0.5-1.5)
	Sleep	3.0^c^ (1.4-6.5)	0.8 (0.4-1.6)
**Perceptions of...**			
	Data review^d^	5.6^c^ (1.7-19.1)	0.5 (0.2-1.4)
	Provider trust of data^d^	1.8 (0.5-6.2)	0.5 (0.1-1.8)
	Patient’s interest in technologies^e^	7.6 (0.9-67.9)	1.8 (0.3-10.5)
	Security and liability^e^	1.6 (0.8-3.3)	1.3 (0.6-2.6)
	Perceived patient barriers^e^	2.5 (0.5-13.3)	1.0 (0.2-4.4)

^a^Reference group is nonusers.

^b^Reference group is advanced practice registered nurses.

^c^Statistically significant by 95% CI.

^d^Reference group is agreement.

^e^Reference group is disagreement.

Overall, providers found review of data to be useful and data to be trustworthy. Providers also perceived that their patients were interested in using these technologies. Security and liability issues with use of data and technologies were not perceived to be barriers to use, but there were concerns about barriers to patient use.

### Provider Recommendations

The majority of the providers had recommended these technologies to their patients for physical activity (124/189, 65.6%) and diet (118/189, 62.4%). Additionally, providers who were technology users themselves were 6.4 times more likely to recommend devices and apps to their patients for physical activity tracking, 6.5 times more likely for diet tracking, and 3.0 times more likely for sleep tracking than nonusers. Users were also 5.6 times more likely than nonusers to perceive these technologies as useful in data review for their patients. We found no significant differences between physicians and APRNs (Tables 3 and 4).

### Perceptions of Usefulness

The providers perceived varying levels of usefulness of these technologies for specific issues, rated on 5-point scales from “not at all useful” to “extremely useful.” Many of the providers thought that these technologies were very useful or extremely useful for tracking physical activity (103/185, 55.6%), tracking diet (91/185, 49.2%), tracking vital signs (80/183, 43.7%), and goal setting (79/183, 43.2%; Table 5). However, the providers perceived that these technologies were not at all or slightly useful for sleep (92/181, 50.8%), smoking (94/178, 52.8%), mental states (108/182, 59.3%), and alcohol or drug use (110/176, 62.5%).

**Table 5:** Perceived usefulness of technologies (N=189).

	Not at all or slightly useful, n (%)	Neutral, n (%)	Very or extremely useful, n (%)
Physical activity (n=185)	22 (12.4)	60 (32.5)	103 (55.1)
Diet (n=185)	27 (16.8)	64 (35.2)	91 (48.0)
Vital signs (n=183)	49 (26.3)	54 (29.8)	80 (43.9)
Goal setting (n=183)	31 (18.0)	73 (39.3)	79 (42.7)
Medication adherence (n=182)	67 (34.5)	56 (31.5)	59 (34.0)
Smoking (n=178)	94 (52.0)	56 (30.0)	28 (18.0)
Sleep (n=181)	92 (50.2)	60 (32.8)	29 (16.9)
Alcohol or drug use (n=176)	110 (61.9)	43 (23.4)	23 (14.7)
Mental states (n=182)	108 (59.1)	55 (29.1)	19 (11.8)

## Discussion

### Principal Findings

This study examined health care providers’ perceptions of consumer-grade, off-the-shelf behavioral tracking technologies. The responses of physicians and APRNs were almost the same. Overall, providers who personally owned or used these technologies were more likely to recommend them for tracking of physical activity, diet, and sleep. Additionally, providers who used these types of technologies were more likely to see the data as useful. Overwhelmingly, the providers who had personal experiences recommended these devices to their patients, indicating that once providers see and understand the data the technologies can provide, they can better counsel their patients in how these devices can help in lifestyle behavior change. We found only one difference between physicians and APRNs in their perceptions of the technologies, pertaining to connections to the patient portal.

Our study demonstrated that many providers strongly agreed or agreed that these technologies have benefits for their patients. Results revealed perceptions of worthwhile benefits to reviewing patient-tracked health data and that data could help in managing patient visits. Past research suggested that patient-generated health data may lead to better communication between the patient and provider, help set goals, and discover patients’ habits and preferences [27]. Additionally, some studies showed that, when patients brought information to their visits, better health outcomes were achieved [25,38,39].

Overall, providers had positive perceptions of trusting the data that these devices provide. In contrast, previous research contended that physicians perceived the data from these devices to be unreliable [40]. However, Nundy et al found that, overall, these data are more trustworthy than self-report, with providers perceiving that some patients misrepresent their activity to please the providers [27]. In previous research regarding health technologies, Health Insurance Portability and Accountability Act of 1996 rules and regulations regarding data security were major concerns among providers [26,28,29]. However, we found that most providers were not concerned with these issues. Yet, if these data were synthesized and standardized for inclusion into the medical record, data security could become more of a concern [26]. Given, that the data do not currently become part of the medical record, there is very limited liability for the providers regarding these data. This also may be because providers are looking at the data as supplemental, as a way to help their patients increase healthy behaviors [41,42]. However, technology is going to continue be to marketed to consumers rather than to primary care providers. These entities—providers, insurers, and regulators—are going to have to consider the infrastructure of information, data infrastructure, and policies concerning data security and privacy as more and more of these technologies are introduced.

Prior work has demonstrated that providers perceive patients’ lack of access to these technologies as a barrier to recommending them to patients [26-29], and our results support these findings. Our research demonstrated that provider-perceived barriers to recommending these technologies included older patients, technical literacy, and financial costs. However, the costs of these technologies are decreasing, ease of use is improving for certain populations, and some insurance companies may try to cover costs for these types of technologies [17].

We found no major differences in perceptions of these technologies between physicians and APRNs. While, to our knowledge, no previous studies have compared perceptions of these technologies between different types of health care providers, some studies examined perceptions of different health-related technologies (eg, telemedicine or EMRs). Physicians tended to be concerned with costs and perceived productivity [27,28,31], whereas nurses were concerned with how much effort went into learning the technologies and the support available for their integration and use in practice [31]. This supports APRNs wanting to link these data to the patient portal. Previous studies have found differences between physicians and APRNs [43]. Future research should examine whether these differences exist within organizations to explore whether differences emerge as these technologies become more commonplace.

Overall, the providers perceived these technologies to be the most useful for tracking physical activity, diet, vital signs, and goal setting. They viewed these technologies not to be effective for monitoring sleep, which is one of the benefits of using many of these consumer devices and apps. This could be because many devices have sleep tracking as a secondary function [44]. The providers also perceived technologies as not being useful for tracking mental states, alcohol or drug use, and smoking, which could be of concern, since some projects are seeking to use mobile devices to help with these areas [45-47]. We surveyed only primary care and family practice health care providers, which could be a potential reason for the perceived lack of usefulness for tracking mental states, alcohol or drug use, and smoking, as some of these issues could be referred to specialists. However, some patients may see only a primary care or family practice health care provider, and they consult with them for all health issues.

### Limitations

This study had several limitations. First, as with any survey, recall bias may have been a factor. Second, this was a survey of physicians and APRNs in Michigan; this study should be replicated to include a US national sample of providers, including a broader range of providers. Third, the response rate was low (33.6%), but previous research has shown a downward trend of response rates when surveying health care providers [48]. Other studies have demonstrated that, in general, most survey respondents are more likely to participate if they are interested in the topic [49]. Fourth, our sample was majority white and women, who have been shown in past research to participate more in surveys [50-53]. Fifth, to ensure that the health care providers understood the class of technologies we were interested in, we asked them specifically about physical activity, diet, and sleep. This may have lowered the perceived usefulness of other ways in which these technologies can be used. However, the technologies used for the other issues we asked them about are in themselves not as popular or well known. As this sector of health care continues to grow, future research should examine these areas in more depth.

### Conclusion

Our survey results have implications for providers, technology developers, patients, and insurers. Once providers have first-hand experience with technologies, they understand how to interpret the data better. Technology developers and manufacturers should continue to test the validity and reliability of their devices and apps to provide the credibility that providers expect. Our results also demonstrated that, if insurers would provide reimbursement for these types of technologies, the cost barrier could be reduced. On the other hand, payers also need to reimburse the providers for time to review the data. Additionally, being able to access these data through the EMR is perceived as an effective way to view the data. This does reinforce the finding that there must be a way to incorporate the data into routine medical encounters. For patients, this could be a way to communicate about their health care preferences and priorities to their provider. Makers of these devices may want to consider advertising directly to providers. Our findings suggested that those providers who already own a device or use a technology were more likely to recommend them to their patients and found positive outcomes through patient use.

This work demonstrated that primary care physicians and APRNs have an overall positive perception of consumer-grade off-the-shelf technologies that track individual health behaviors. Providers may serve as the gatekeepers for use of these technologies in improving health care, as their actual use could drive interest, acceptance, and possible better health outcomes. These providers, as trusted sources of health information, could be advocates for use of these behavioral health tracking technologies and help realize the public health benefits that could come from their wider adoption.

## Abbreviations

APRN: advanced practice registered nurse
EMR: electronic medical record system
